# A transfer learning-based hybrid deep- and machine-learning regression approach for predicting the postoperative pulmonary function

**DOI:** 10.1186/s41747-026-00821-z

**Published:** 2026-09-30

**Authors:** Wenfang Wang, Yingli Sun, Haihong Ma, Ming Li

**Affiliations:** 1 Department of Radiology, Huadong Hospital, Fudan University, 200040 Shanghai, People’s Republic of China; 2 Department of Radiology, Renji Hospital, School of Medicine, Shanghai Jiao Tong University, 200127 Shanghai, People’s Republic of China; 3 Kashi Prefecture Second People’s Hospital, 844000 Xinjiang, People’s Republic of China; 4 Department of Radiology, Shanghai Chest Hospital, School of Medicine, Shanghai Jiao Tong University, 200030 Shanghai, People’s Republic of China

**Keywords:** Deep learning, Regression analysis, Respiratory function tests, Tomography (x-ray computed), Transfer machine learning

## Abstract

**Objectives:**

Accurately predicting postoperative pulmonary function is essential for surgical decision-making in patients with pulmonary nodules. Here, we developed a hybrid model using preoperative computed tomography (CT) images and evaluated its accuracy and interpretability for predicting postoperative pulmonary function.

**Materials and methods:**

This retrospective study included 136 patients who underwent preoperative chest CT and postoperative pulmonary function tests. A pre-trained Inflated 3D ConvNet (I3D) model was fine-tuned for transfer learning to predict the postoperative pulmonary function, and the resulting model served as the feature extractor. Extracted deep learning features were combined with an elastic net regression for prediction. Models established using clinical features or radiomics with elastic net, end-to-end I3D transfer learning, and 3D ResNet18 trained from scratch were compared, with additional evaluation against a conventional segment-counting method. Performance was evaluated against spirometry, and interpretability was assessed using Grad-CAM.

**Results:**

The hybrid model exhibited the best external test performance. For postoperative forced vital capacity (FVC) prediction, the concordance correlation coefficient (CCC) was 0.707, the Pearson correlation coefficient (Pearson *r*) was 0.796, and the *R*-squared (*R*^2^) was 0.499. For postoperative forced expiratory volume in 1 s (FEV_1_) prediction, the CCC was 0.729, Pearson *r* was 0.772, and *R*^2^ was 0.418. Grad-CAM revealed the inferior and paravertebral lung regions in the FVC model and bilateral lung bases and diaphragmatic areas in the FEV_1_ model.

**Conclusion:**

The hybrid elastic net model based on fine-tuned I3D features predicted postoperative pulmonary function without requiring preoperative spirometry or detailed surgical planning and may guide the development of future predictive models.

**Key Points:**

***Question*** CT-only prediction of postoperative pulmonary function remains an unmet clinical need due to the non-routine use of pulmonary function testing.

***Findings*** A hybrid CT-based model enables prediction of postoperative pulmonary function and outperforms conventional segment-counting, clinical, radiomics, and end-to-end deep learning approaches.

***Relevance Statement*** This hybrid CT-based model can estimate postoperative pulmonary function from routine preoperative non-contrast CT images, potentially assisting surgical decision-making for lung cancer.

**Graphical Abstract:**

**Figure:**
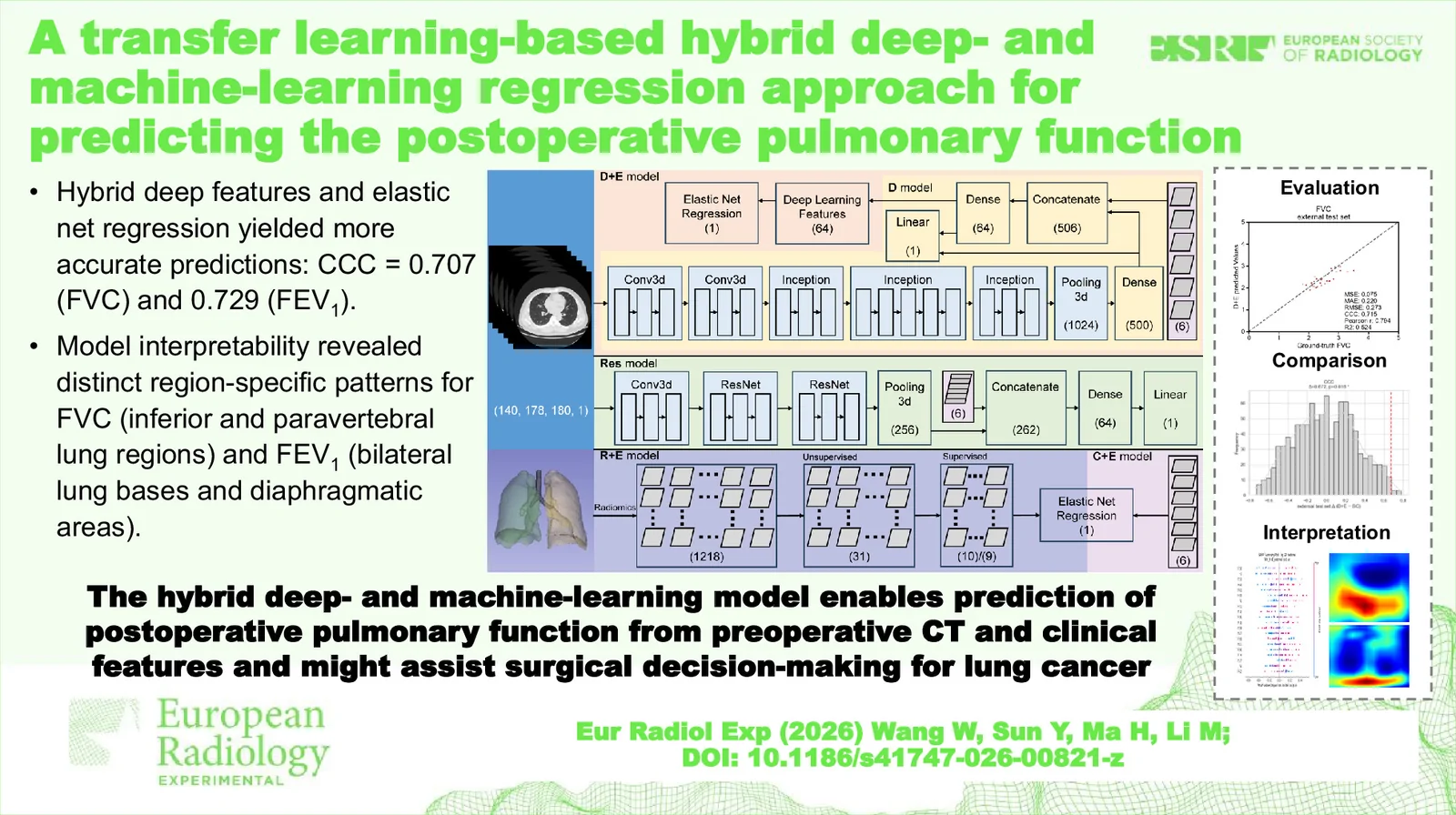

## Background

Lung cancer remains the leading cause of cancer-related mortality and imposes a substantial global health burden [1]. Surgical resection is widely considered the most effective treatment for lung cancer [2–4]. Advances in imaging have increased the incidental detection of pulmonary nodules, now reported in 20–31% of chest computed tomography (CT) examinations, and the volume of lung surgery has risen accordingly [5–9]. Greater emphasis on postoperative quality of life has increased patient and clinician attention to postoperative pulmonary function loss, which relates closely to postoperative complications and recovery [10–12]. Lobectomy, segmentectomy, or wedge resection is typically performed based on the nodule size and tumor characteristics to preserve the functional lung parenchyma while maintaining oncologic adequacy [13–15]. Accurately predicting postoperative pulmonary function has become a central challenge in surgical planning and shared decision-making.

Clinicians have traditionally estimated postoperative pulmonary function using the segment-counting method, which predicts functional loss from the number of lung segments resected [16–18]. CT- or positron emission tomography/CT-based volumetric analyses have also been used to assess the amount of lung tissue to be removed [19–24]. However, these approaches generally apply only to lobectomy or segmentectomy and do not suit wedge resection, where the resection volume is smaller than the segment. They also rely on preoperative pulmonary function as a baseline and often overestimate functional loss after anatomical lung resections [17, 18]. Consequently, clinicians require a predictive method that applies to a wider range of surgical procedures.

Deep learning models have automatically learned hierarchical feature representations through multiple layers of nonlinear transformations [25]. Each successive layer captures increasingly abstract features, and the final layer often functions as a linear classifier built on the rich features extracted by the preceding layers. Using these learned features as inputs for traditional machine learning models, such as linear regression or support vector machines, can enhance predictive performance, especially with limited data [26–28]. To circumvent the need for extensive data and computational resources for training deep models, pre-trained networks can be used for transfer learning, providing readily accessible and informative feature representations [29–31].

In this study, we developed a hybrid pipeline that combines transfer-learned deep features with a regularized regression model to predict the postoperative pulmonary function using preoperative CT. Specifically, we used an Inflated 3D ConvNet (I3D) pre-trained for pulmonary function assessment as a feature extractor and fed the extracted representations into an elastic net regression model to estimate the primary outcome (*e.g*., forced vital capacity (FVC) and forced expiratory volume in 1 s (FEV_1_)). We hypothesized that (i) integrating pre-trained 3D imaging features with an elastic net would enable accurate prediction across lobectomy, segmentectomy, and wedge resection, and (ii) this hybrid strategy would perform at least comparably to conventional segment-counting, clinical, radiomics, end-to-end transfer learning, and models trained from scratch. Our results aim to support individualized surgical decision-making by providing a method applicable to common resection types.

## Materials and methods

### Study population

This retrospective study was approved by the Institutional Review Board of Huadong Hospital, affiliated to Fudan University (No. 20220026), and the requirement for written informed consent was waived. Data from 7,494 patients who underwent pulmonary function tests (PFTs) were collected from Huadong Hospital (Institution A) between October 2014 and October 2023. Clinical and demographic characteristics, chest CT images, and spirometry reports were reviewed. Six clinical features were included as model inputs: sex, age, height, weight, resection location, and resection type.

Resection locations were classified as left lower lobe, left upper lobe, right lower lobe, right middle lobe, right upper lobe, or involving more than one lobe.

Resection types were classified as wedge resection, segmentectomy, or lobectomy. For patients who underwent multiple procedures during the same surgery, only the most extensive procedure was recorded.

**Fig. 1:**
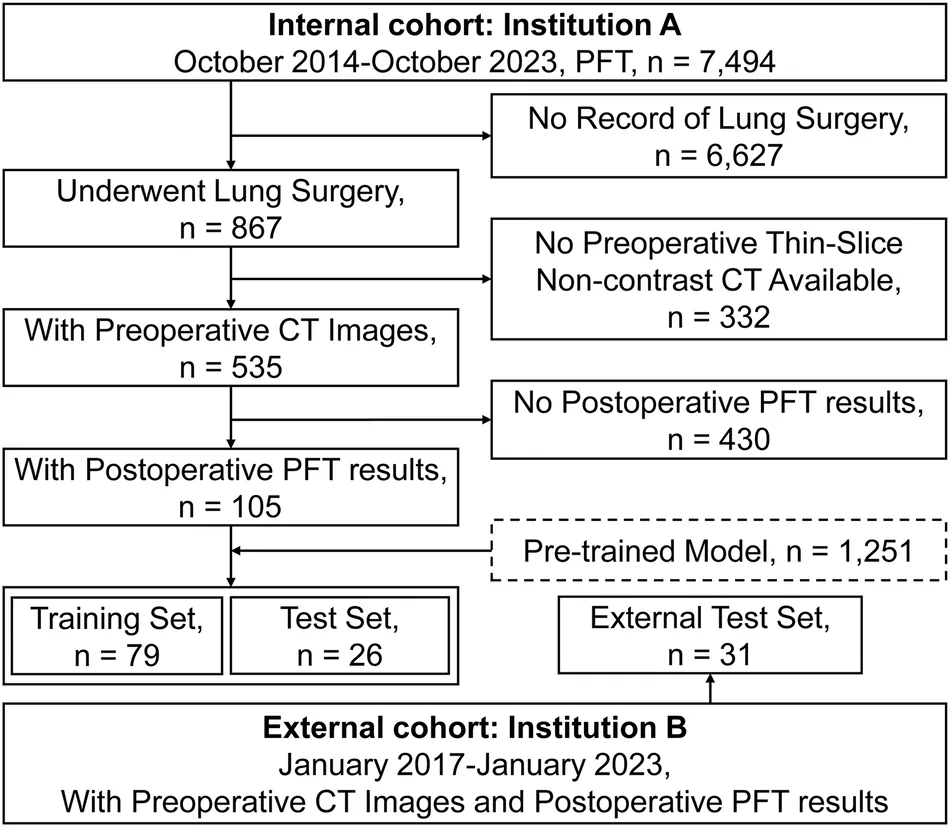
Patient flow diagram including data partitioning. The dataset used to train the pre-trained model was independent of and unrelated to the current study population. CT, Computed tomography; PFT, Pulmonary function test

The exclusion criteria were: no history of lung surgery at Huadong Hospital, no preoperative thin-slice non-contrast CT images or poor-quality images, and no postoperative PFT results. Postoperative PFTs were not routine and were obtained only when clinically indicated, resulting in a nonrandom pattern of missing data. After applying these criteria, we randomly assigned cases to training and test sets (Fig. 1).

An independent external test cohort was obtained from Heilongjiang Provincial Hospital (Institution B) between January 2017 and January 2023. A total of 31 patients who met the same eligibility criteria as the development cohort were included in the external test (Fig. 1).

### Data acquisition

PFTs followed the 2019 American Thoracic Society and European Respiratory Society guidelines and were performed using the Jaeger MasterScreen Pro system [32]. Up to three measurements were obtained for each patient to determine optimal FVC and FEV_1_.

Chest CT scans were acquired using GE Discovery CT750 HD, GE Revolution CT Power (GE Healthcare, Boston, America), Somatom Definition Flash, Somatom Drive, and Somatom Force (Siemens Healthineers, Erlangen, Germany). Scanning parameters were: tube voltage 120 kVp, tube current 100–200 mA, pitch 0.75–1.5, detector collimation 1–1.5 mm, and reconstruction slice thickness 1–1.25 mm. All the images were reconstructed using medium-sharp kernels.

### Segment-counting analysis

For patients in the segmentectomy and lobectomy subgroup who had preoperative PFTs, the predicted postoperative pulmonary function was calculated using the segment-counting method with the following formula [33]:

$$	{{{\rm{predicted}}}} \; {{{\rm{postoperative}}}} \, {FVC} \, {{{\rm{or}}}} \, {{FEV}}_{1} \\ 	\!\!= \!{{{\rm{preoperative}}}}\,{FVC}\,{{{\rm{or}}}}\,{{FEV}}_{1} \times \frac{19-{segment}\,{of}\,{lung}\,{to}\,{be}\,{resected}}{19}$$

The total number of segments in both lungs was 19, as previously described by Olsen and colleagues [34].

### Image preprocessing

All CT images were manually reviewed using the Nibabel package (version 5.1.0) in Python (version 3.9.7), and slices containing lung tissue were selected; slices without visible lung parenchyma were excluded from subsequent analyses.

The selected images were normalized to 12-bit intensity values and resampled to a three-dimensional size of 140 × 178 × 180, for model input. For radiomics feature extraction, the corresponding masks were prepared using the LungCTAnalyzer extension (https://github.com/rbumm/SlicerLungCTAnalyzer/) in 3D Slicer (version 5.4.0; http://www.slicer.org) [35, 36], detailed information is provided in Additional file 1, Method S2.

### Model development

The hybrid transfer-learning pipeline, referred to as **D** + **E**, was developed based on a pre-trained I3D network. The pre-trained model was originally trained to predict pulmonary function indicators from the preoperative CT scans of patients who had not undergone surgery. The pretraining dataset was independent of those used in the present study, with no patient-level overlap. Detailed information regarding the pretraining dataset, training procedure, and model configuration is provided in Additional file 1, Method S1. In the current study, the weights of the pre-trained model were loaded, and the last 15 layers were unfrozen to enable fine-tuning on our training set to predict postoperative pulmonary function indicators. In the fine-tuned model, six standardized clinical features were concatenated to the original penultimate layer (dense, 500 units), followed by a dense layer (64 units) and a linear regression layer (1 unit). To mitigate potential data leakage, transfer learning was performed for over 50 epochs using five-fold cross-validation on the training set. The model was implemented in Keras and TensorFlow (version 2.8.0) using Python and trained on an NVIDIA GeForce RTX 3060 (12 GB) GPU.

The resulting models were used as feature extractors, from which the 64-dimensional representations of the penultimate layer were obtained for each fold. The out-of-fold features across the five folds served as the final feature representation and were subsequently used to train an elastic net regression model to predict postoperative pulmonary function indicators. Elastic net hyperparameters were optimized using 10-fold cross-validation on the training set to determine the optimal regularization parameters (*α* and *λ*), and model performance was evaluated on the test set.

**Fig. 2:**
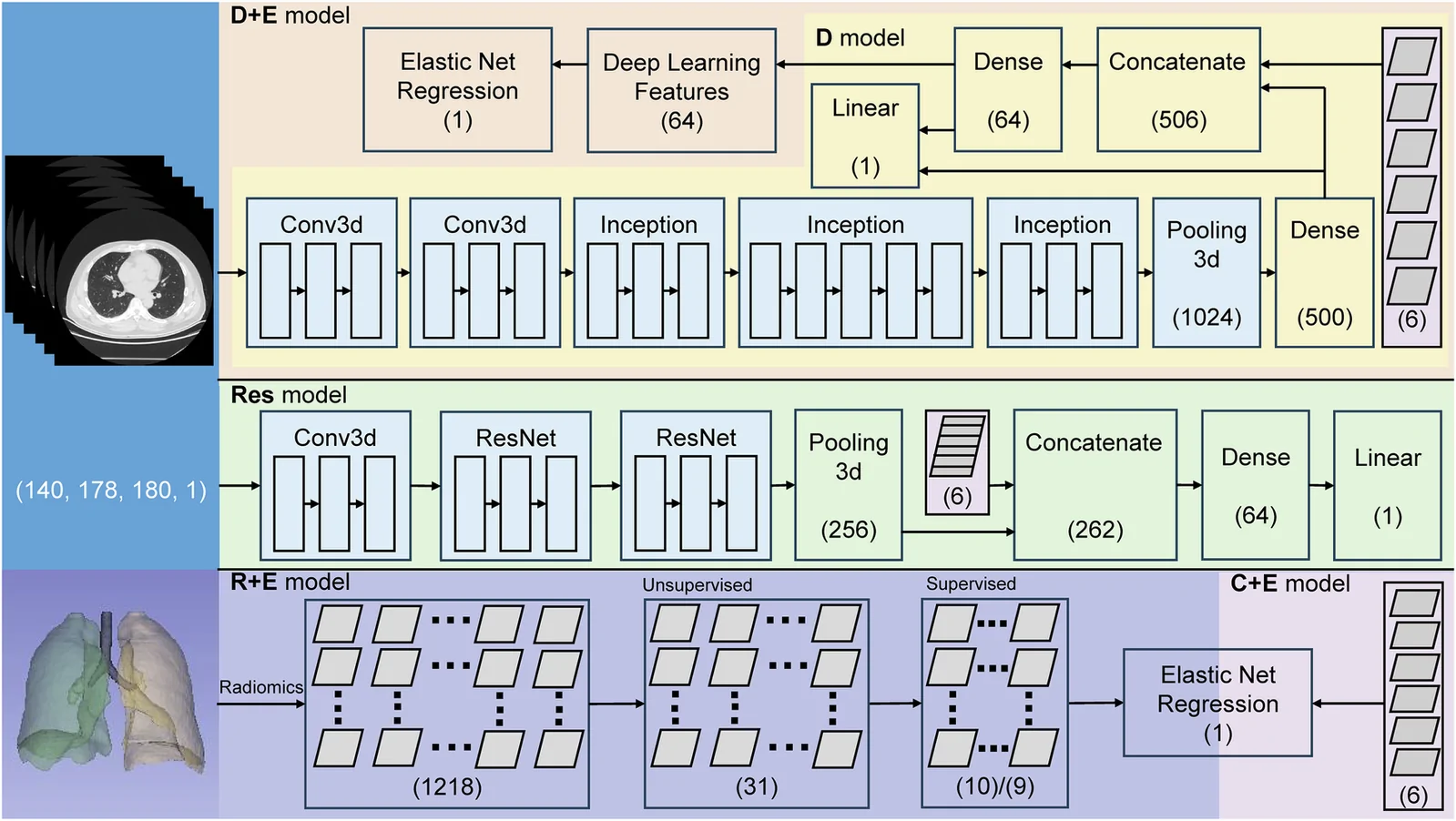
Schematic diagram of the study workflow with a comparison of the five model architectures. The **D** + **E**, **D**, **Res**, **R** + **E**, and **C** + **E** models appear against light orange, light yellow, light green, purple, and pale purple backgrounds, respectively. The deep learning backbone is shown in light blue, and the clinical features are shown in pale purple

For comparison, four additional models were developed using identical training data, evaluated on an internal test set, and their performance was further validated on an independent external test set (Fig. 2).

1. Radiomics + Elastic Net (**R** + **E**) using the radiomics features shown in Additional file 1, Supplementary Table S1.
2. Clinical features + Elastic Net (**C** + **E**).
3. 3D ResNet18 (**Res**).
4. Fine-tuned I3D without elastic net (**D**).

Minor preprocessing or architecture-specific adjustments were applied to the comparator models as required. The full configuration and preprocessing details for all the models are provided in Additional file 1, Methods S2–3.

### Model interpretability

We used two complementary approaches to interpret the model's predictions. The most salient regions in the CT images that influenced predictions based on deep features were highlighted with Grad-CAM, providing a spatial understanding of the model's decision-making [37]. SHapley Additive exPlanations (SHAP) was applied to quantify the contribution of individual features, with the top 20 features visualized to illustrate their impact on model outputs [38].

### Statistical analysis

All data were analyzed using Python. All tests were two-tailed, and *p* < 0.05 was considered statistically significant.

Continuous and categorical variables among the three groups were compared using Kruskal–Wallis and chi-square tests. The agreement between spirometry-measured PFT values and those predicted by the models was evaluated using the mean absolute error (MAE), mean squared error (MSE), root mean square error (RMSE), Pearson correlation coefficient (Pearson *r*), concordance correlation coefficient (CCC), and *R*-squared (*R*^2^) values. Bland–Altman plots were analyzed to estimate bias and 95% limits of agreement. To estimate the uncertainty of these metrics, 1,000 bootstrap resamples of the test set were generated, and mean values and 95% confidence intervals were calculated for each metric. Pairwise comparisons between models were conducted using a nonparametric permutation test with 1,000 iterations. The *p*-value was defined as the proportion of permuted performance differences that exceeded the observed difference. Paired comparisons of absolute errors between models were assessed using Wilcoxon signed-rank tests. The original metrics calculated for the full and external test sets might have slightly differed from the bootstrap mean values, owing to sampling variability. Additionally, FVC%pred and FEV_1_%pred were used to derive binary classifications with a clinically relevant threshold of 80%. Receiver operating characteristic analysis was performed to evaluate the model's discriminative performance at this threshold. Model performance was quantified using the area under the receiver operating characteristic curve (AUC), sensitivity, specificity, and positive and negative predictive values.

## Results

### Clinical characteristics

Of the 7,494 participants recruited, 105 (61 females and 44 males) met the inclusion criteria and were enrolled (Fig. 1). All patients underwent lung resection at Huadong Hospital and had preoperative thin-section non-contrast CT scans and postoperative PFT reports available. The mean age of the cohort was 65.4 years (range, 32–80 years). The participants were randomly assigned to the training and test sets in a 0.75:0.25 ratio. In addition, an independent external test set consisting of patients from Heilongjiang Provincial Hospital (*n* = 31; 22 females; mean age, 60.3 years; range, 29–76 years) was used to evaluate model generalizability.

The surgical history of each patient was thoroughly reviewed. Based on the operative records, two categorical variables, resection location and resection type, were documented (Table 1). All variables presented in Table 1, which comprise six clinical characteristics and PFT parameters, were compared among the training, test, and external test sets. Most variables did not significantly differ among the three groups (all *p* > 0.05), except for FEV_1_%pred < 80% (*p* = 0.002) and FEV_1_/FVC (%) (*p* = 0.013). These variables were retained owing to their clinical relevance.

**Table 1:** Patient characteristics in different data sets

Variables	Training *n* = 79	Test *n* = 26	External test *n* = 31	*p*-value
Age, years	65.3 ± 9.24	65.5 ± 7.45	60.3 ± 12.3	0.123
Female	45, 57.0%	16, 61.5%	22, 71.0%	0.399
Height, mm	164 ± 7.93	164 ± 9.10	162 ± 7.13	0.573
Weight, kg	63.9 ± 9.06	62.6 ± 10.8	63.2 ± 11.2	0.548
Resection type				0.078
Wedge resection	35, 44.3%	12, 46.2%	7, 22.6%	
Segmentectomy	16, 20.3%	4, 15.4%	13, 41.9%	
Lobectomy	28, 35.4%	10, 38.5%	11, 35.5%	
Resection location				0.120
More than 1 lobe	12, 15.2%	3, 11.5%	8, 25.8%	
Left lower lobe	15, 19.0%	2, 7.7%	5, 16.1%	
Left upper lobe	19, 24.1%	7, 26.9%	3, 9.68%	
Right lower lobe	5, 6.33%	4, 15.4%	7, 22.6%	
Right middle lobe	6, 7.59%	0, 0%	1, 3.23%	
Right upper lobe	22, 27.8%	10, 38.5%	7, 22.6%	
FVC, L	2.35 ± 0.556	2.28 ± 0.514	2.49 ± 0.403	0.184
FVC%pred, < 80%	17, 21.5%	5, 19.2%	11, 35.5%	0.246
FEV_1_, L	1.98 ± 0.491	1.90 ± 0.392	1.97 ± 0.350	0.582
FEV_1_%pred, < 80%	14, 17.7%	4, 15.4%	15, 48.4%	0.002**
FEV_1_/FVC (%)	84.4 ± 11.0	84.3 ± 9.16	79.2 ± 9.02	0.013*
FEV_1_/FVC (%), < 70%	2, 2.53%	0, 0%	0, 0%	0.481

Categorical variables were reported as the number of cases and percentage, whereas continuous variables were expressed as mean ± standard deviation. Values are rounded to three significant digits

*FEV*_1_ Forced expiratory volume in 1 s, *FVC* Forced vital capacity

* *p* < 0.05; ** *p* < 0.01

### Model performance

**Fig. 3:**
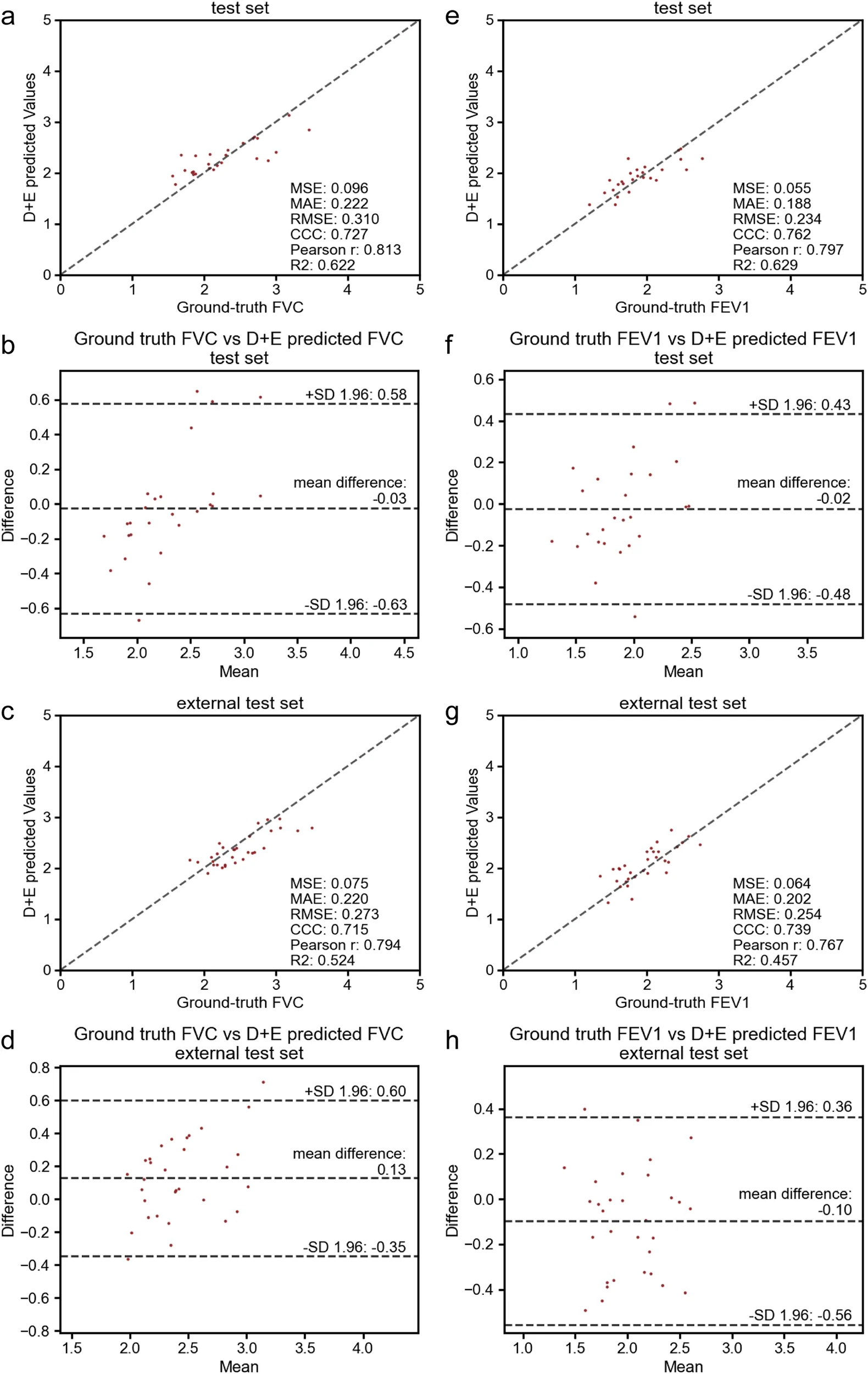
Performance of the **D** + **E** model for (**a**–**d**) FVC and (**e**–**h**) FEV_1_. **a**, **c**, **e**, and **g** Scatterplots indicating the agreement between ground-truth values and model-predicted values. The diagonal lines represent the ideal predictions. **b**, **d**, **f**, and **h** Bland–Altman plots indicating bias and ± 95% limits of agreement (indicated by dashed lines). CCC, Concordance correlation coefficient; FEV_1_, Forced expiratory volume in 1 s; FVC, Forced vital capacity; MAE, Mean absolute error; MSE, Mean squared error; Pearson *r*, Pearson correlation coefficient; *R*^2^, *R*-squared; RMSE, Root mean squared error; SD, Standard deviation

The performance of the five regression models (**D** + **E**, **R** + **E**, **C** + **E**, **Res**, and **D**) on the test and external test sets is presented in Tables 2 and 3, respectively. The **D** + **E** model consistently achieved the best overall performance on both the test and external test sets, with particularly strong results for the external test set, including the lowest prediction error (MSE = 0.074 L^2^, 0.065 L^2^; MAE = 0.219 L, 0.202 L; RMSE = 0.271 L, 0.253 L for FVC and FEV_1_, respectively) and the highest correlation and agreement with the observed values (*R*^2^ = 0.499, 0.418; Pearson *r* = 0.796, 0.772; CCC = 0.707, 0.729 for FVC and FEV_1_, respectively). Figure 3 shows a detailed visualization of the **D** + **E** model for the test and external test sets.

**Table 2:** Performance measures of models in the test set for predicting FVC and FEV_1_

Performance parameters	Test set				
	D + E	R + E	C + E	*D*	Res
**FVC**					
CCC	0.704 [0.498, 0.856]	0.390 [-0.012, 0.683]	0.419 [0.280, 0.561]	0.303 [0.114, 0.536]	0.177 [-0.022, 0.343]
Pearson *r*	0.803 [0.613, 0.918]	0.412 [-0.013, 0.712]	0.794 [0.625, 0.911]	0.536 [0.259, 0.780]	0.378 [-0.043, 0.706]
*R*^2^	0.589 [0.319, 0.779]	-0.062 [-0.873, 0.434]	0.336 [0.092, 0.494]	0.179 [-0.114, 0.446]	0.081 [-0.283, 0.281]
MSE, L^2^	0.098 [0.046, 0.155]	0.247 [0.131, 0.391]	0.159 [0.096, 0.236]	0.201 [0.091, 0.345]	0.221 [0.132, 0.309]
MAE, L	0.225 [0.143, 0.311]	0.389 [0.276, 0.518]	0.335 [0.253, 0.422]	0.346 [0.239, 0.461]	0.396 [0.297, 0.494]
RMSE, L	0.309 [0.214, 0.394]	0.493 [0.362, 0.625]	0.396 [0.310, 0.486]	0.443 [0.302, 0.587]	0.467 [0.364, 0.556]
**FEV**_**1**_					
CCC	0.745 [0.564, 0.871]	0.535 [0.308, 0.724]	0.428 [0.292, 0.544]	0.177 [0.017, 0.338]	0.070 [-0.128, 0.274]
Pearson *r*	0.795 [0.626, 0.901]	0.564 [0.342, 0.743]	0.840 [0.693, 0.926]	0.347 [0.041, 0.569]	0.133 [-0.285, 0.502]
*R*^2^	0.596 [0.281, 0.784]	0.128 [-0.454, 0.477]	0.334 [0.038, 0.486]	-0.209 [-0.986, 0.168]	-0.073 [-0.507, 0.189]
MSE, L^2^	0.055 [0.029, 0.088]	0.117 [0.080, 0.157]	0.092 [0.057, 0.132]	0.164 [0.109, 0.233]	0.151 [0.087, 0.229]
MAE, L	0.188 [0.139, 0.244]	0.303 [0.243, 0.365]	0.259 [0.200, 0.321]	0.365 [0.301, 0.434]	0.315 [0.234, 0.402]
RMSE, L	0.232 [0.169, 0.297]	0.341 [0.282, 0.396]	0.301 [0.238, 0.363]	0.403 [0.331, 0.482]	0.386 [0.294, 0.479]

Data are expressed as *n* [95% confidence intervals]

*CCC* Concordance correlation coefficient, *FEV*_*1*_ Forced expiratory volume in 1 s, *FVC* Forced vital capacity, *MAE* Mean absolute error, *MSE* mean squared error, *Pearson* *r* Pearson correlation coefficient, *R*^2^ R-squared, *RMSE* root mean squared error

**Table 3:** Performance measures of models in the external test set for predicting FVC and FEV_1_

Performance parameters	External test set				
	D + E	R + E	C + E	*D*	Res
**FVC**					
CCC	0.707 [0.588, 0.812]	0.211 [-0.112, 0.525]	0.411 [0.314, 0.521]	0.128 [-0.0120, 0.249]	0.142 [0.051, 0.264]
Pearson *r*	0.796 [0.692, 0.870]	0.244 [-0.137, 0.558]	0.780 [0.629, 0.884]	0.299 [-0.046, 0.578]	0.367 [0.140, 0.573]
*R*^2^	0.499 [0.251, 0.673]	-0.532 [-1.600, 0.175]	0.214 [-0.051, 0.409]	-1.343 [-3.073, -0.459]	-0.433 [-0.934, -0.033]
MSE, L^2^	0.074 [0.043, 0.115]	0.226 [0.107, 0.366]	0.119 [0.063, 0.192]	0.338 [0.217, 0.478]	0.219 [0.112, 0.351]
MAE, L	0.219 [0.167, 0.281]	0.351 [0.250, 0.468]	0.264 [0.192, 0.344]	0.472 [0.368, 0.585]	0.342 [0.238, 0.458]
RMSE, L	0.271 [0.207, 0.339]	0.470 [0.327, 0.605]	0.342 [0.251, 0.438]	0.578 [0.465, 0.691]	0.464 [0.334, 0.592]
**FEV**_**1**_					
CCC	0.729 [0.599, 0.837]	-0.068 [-0.383, 0.275]	0.322 [0.192, 0.441]	0.332 [0.067, 0.546]	0.134 [-0.084, 0.318]
Pearson *r*	0.772 [0.650, 0.863]	-0.076 [-0.422, 0.326]	0.588 [0.368, 0.768]	0.434 [0.087, 0.714]	0.280 [-0.164, 0.633]
*R*^2^	0.418 [0.043, 0.659]	-0.868 [-2.031, -0.036]	0.236 [0.032, 0.367]	0.123 [-0.372, 0.426]	-0.017 [-0.395, 0.242]
MSE, L^2^	0.065 [0.039, 0.090]	0.210 [0.108, 0.327]	0.088 [0.057, 0.125]	0.099 [0.057, 0.145]	0.115 [0.071, 0.163]
MAE, L	0.202 [0.146, 0.254]	0.361 [0.259, 0.471]	0.244 [0.188, 0.304]	0.249 [0.181, 0.318]	0.265 [0.193, 0.338]
RMSE, L	0.253 [0.197, 0.300]	0.455 [0.328, 0.572]	0.294 [0.238, 0.353]	0.312 [0.238, 0.381]	0.337 [0.267, 0.403]

Data are expressed as *n* [95% confidence intervals]

*CCC* concordance correlation coefficient, *FEV*_1_ forced expiratory volume in 1 s, *FVC* forced vital capacity, *MAE* mean absolute error, *MSE* mean squared error, *Pearson* *r* Pearson correlation coefficient, *R*^2^ *R*-squared, *RMSE* root mean squared error

In addition to the **D** + **E** model, the remaining four models demonstrated varying levels of predictive performance across the test set and the external test set. The performance of the **C** + **E** model was comparable to that of the **D** + **E** model in terms of Pearson *r* in both datasets (external test set: Pearson *r* = 0.780 and 0.588 for FVC and FEV_1_, respectively). However, the CCC and *R*^2^ were consistently lower (external test set: CCC = 0.411, 0.322; *R*^2^ = 0.214, 0.236 for FVC and FEV_1_, respectively), indicating reduced agreement in absolute values despite the preserved correlation. The **R** + **E** model showed moderate performance in the test set but a pronounced decline in FEV_1_ in the external test set (CCC decreased from 0.535 to -0.068), whereas a smaller reduction was observed for FVC (CCC decreased from 0.390 to 0.211), reflecting limited generalizability across datasets. The **D** model showed limited predictive ability, with a Pearson *r* of 0.299 for FVC and 0.434 for FEV_1_ in the external set, indicating that **D** features alone are insufficient for accurate prediction. By contrast, the **Res** model was the weakest across most outcomes, with consistently inferior results compared with the other models in both datasets, indicating minimal learning from the available features.

Pairwise comparisons between models were conducted using a nonparametric permutation test with 1,000 iterations (Tables 4 and 5). In the test set, the **D** + **E** model performed significantly better than most competing models among evaluation metrics, particularly for CCC, and only limited nonsignificant comparisons were evident (such as *p* = 0.058 for the **R** + **E** model in FEV_1_ prediction). The overall superiority of the **D** + **E** model remained consistent in the external test set, although the statistical significance of several pairwise comparisons was attenuated, particularly for MAE and RMSE when compared with the **C** + **E**, **D**, and **Res** models. Despite this reduction, the **D** + **E** model maintained the most favorable overall performance among the metrics, supporting its generalizability under external validation.

**Table 4:** Results of permutation tests for regression models on the test set predicting FVC and FEV_1_

Performance parameters	Test set							
	D + E *vs*. R + E		D + E *vs*. C + E		D + E *vs*. D		D + E *vs*. Res	
	Difference	*p-*value	Difference	*p-*value	Difference	*p-*value	Difference	*p-*value
**FVC**								
CCC	0.289	0.045*	0.291	< 0.001***	0.420	0.004**	0.294	0.025*
MAE, L	-0.163	< 0.001***	-0.112	< 0.001***	-0.122	0.001**	-0.113	0.004**
RMSE, L	-0.183	< 0.001***	-0.088	0.001**	-0.138	0.006**	-0.091	0.046*
**FEV**_**1**_								
CCC	0.191	0.058	0.320	0.021*	0.579	0.001**	0.374	0.003**
MAE, L	-0.113	0.003**	-0.071	0.048*	-0.178	< 0.001***	-0.076	0.039*
RMSE, L	-0.106	0.007**	-0.070	0.094	-0.173	< 0.001***	-0.086	0.018*

*CCC* Concordance correlation coefficient, *FEV*_1_ Forced expiratory volume in 1 s, *FVC* Forced vital capacity, *MAE* Mean absolute error, *RMSE* Root mean squared error

* *p* < 0.05; ** *p* < 0.01; *** *p* < 0.001

**Table 5:** Results of permutation tests for regression models on the external test set predicting FVC and FEV_1_

Performance parameters	External test set							
	D + E *vs*. R + E		D + E *vs*. C + E		D + E *vs*. D		D + E *vs*. Res	
	Difference	*p*-value	Difference	*p-*value	Difference	*p*-value	Difference	*p*-value
**FVC**								
CCC	0.497	0.004**	0.298	0.001**	0.580	< 0.001***	0.573	< 0.001***
MAE, L	-0.131	0.012*	-0.044	0.137	-0.257	< 0.001***	-0.121	0.006**
RMSE, L	-0.203	< 0.001***	-0.072	0.031*	-0.313	0.001**	-0.194	0.001**
**FEV**_**1**_								
CCC	0.818	< 0.001***	0.408	0.001**	0.392	0.021*	0.601	0.001**
MAE, L	-0.158	0.004**	-0.041	0.269	-0.047	0.321	-0.064	0.164
RMSE, L	-0.204	0.003**	-0.041	0.229	-0.060	0.260	-0.086	0.048*

*CCC* Concordance correlation coefficient, *FEV*_1_ Forced expiratory volume in 1 s, *FVC* Forced vital capacity, *MAE* Mean absolute error, *RMSE* Root mean squared error

* *p* < 0.05; ** *p* < 0.01; *** *p* < 0.001

**Fig. 4:**
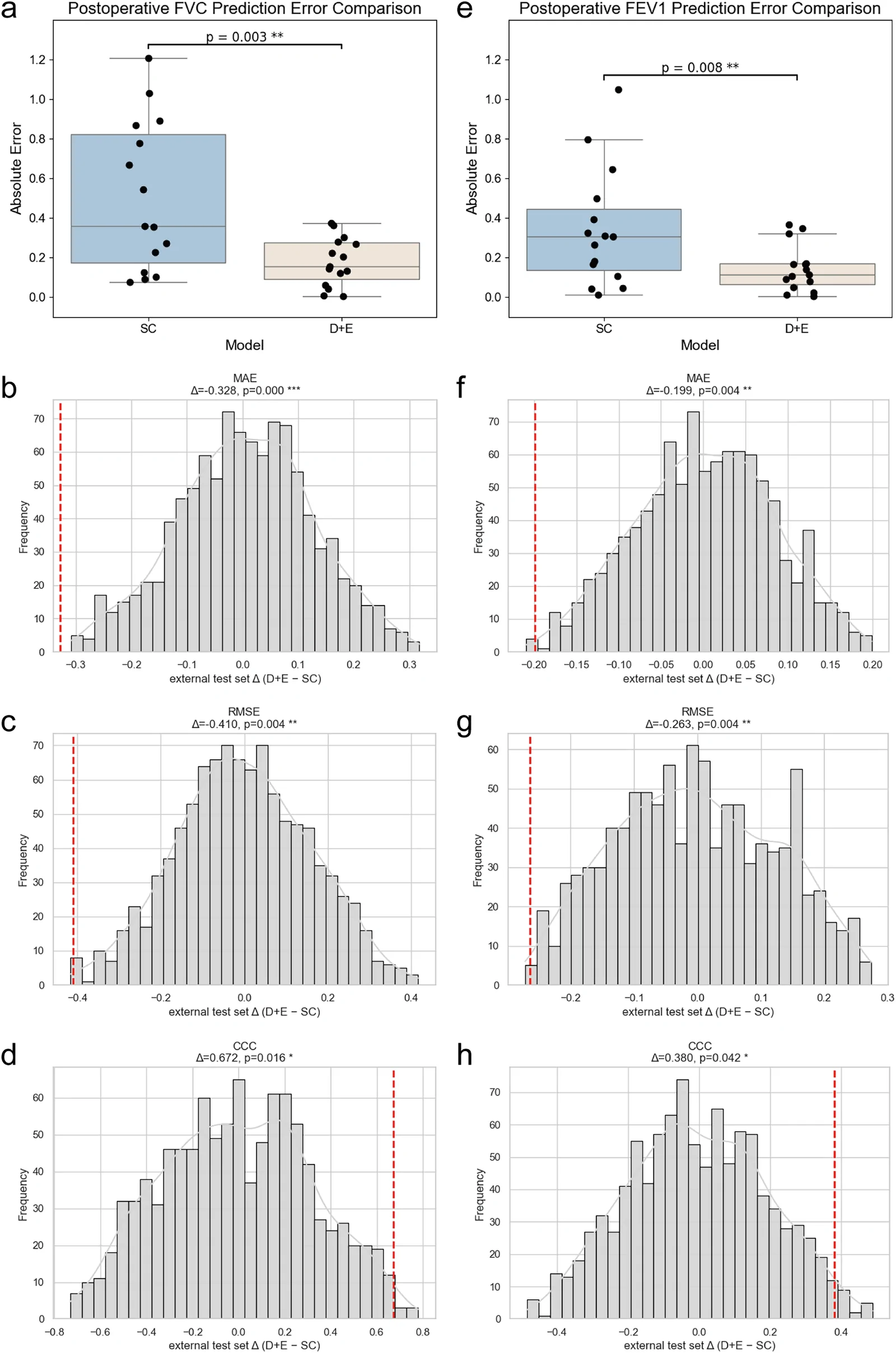
Comparison of prediction performance for FVC and FEV_1_ between SC and **D** + **E** models. **a** (FVC) and **e** (FEV_1_): boxplots of absolute errors, with individual data points overlaid; *p*-values were calculated using the Wilcoxon signed-rank test. **b**–**d** (FVC) and **f**–**h** (FEV_1_): Permutation test plots show results for MAE, RMSE, and CCC, respectively. The red vertical dashed line indicates the observed performance difference between the models, whereas the histograms represent null distributions generated by randomly permuting model labels across paired samples. * *p* < 0.05; ** *p* < 0.01; *** *p* < 0.001. CCC, Concordance correlation coefficient; FEV_1_, Forced expiratory volume in 1 s; FVC, Forced vital capacity; MAE, Mean absolute error; RMSE, Root mean squared error

In the external test set, patients who underwent segmentectomy or lobectomy and had available preoperative pulmonary function results were used to predict postoperative pulmonary function using segment-counting analysis. For both FVC and FEV_1_, the hybrid model (**D** + **E**) consistently outperformed the conventional segment-counting method (SC), with significantly lower errors and closer MAE, RMSE, and CCC agreement based on permutation tests and reduced absolute errors confirmed by Wilcoxon signed-rank tests (Fig. 4).

We assessed the potential clinical utility of threshold-based decision-making, using an exploratory analysis with a predicted clinically relevant cutoff of 80% (Additional file 1, Supplementary Table S2). In the external test set, the **D** + **E** model achieved an AUC of 0.745 for FVC%pred and 0.758 for FEV_1_%pred. The FVC%pred in this model revealed high sensitivity (90.9%) for identifying patients with impaired pulmonary function, whereas FEV_1_%pred revealed high specificity (87.5%).

### Model interpretation

**Fig. 5:**
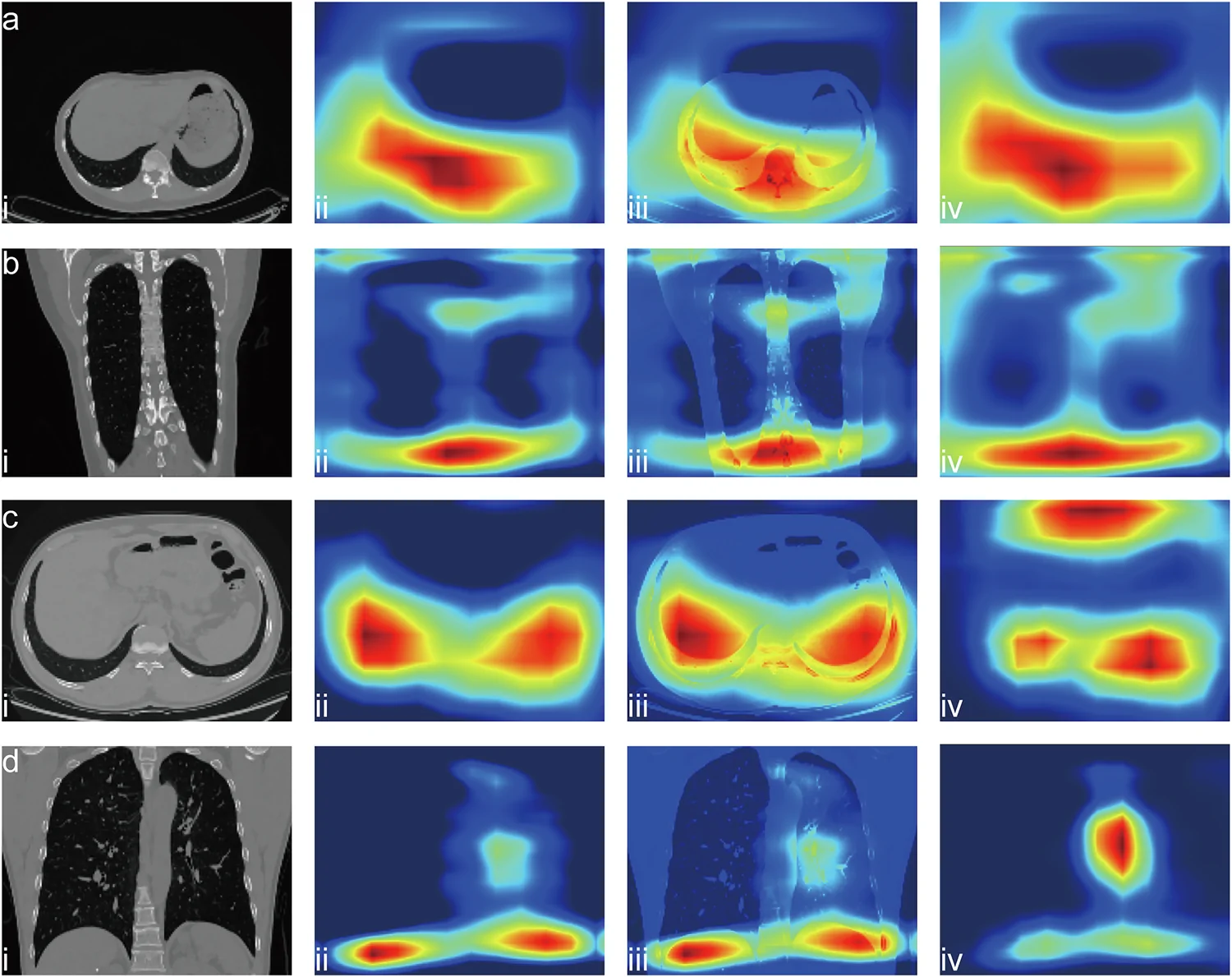
Model interpretation using Grad-CAM visualizations for (**a**, **b**) FVC and (**c**, **d**) FEV_1_. Representative patients were selected to assess each outcome. FVC: a female patient underwent segmentectomy of the lingular segment of the left upper lobe for invasive adenocarcinoma, with a ground-truth FVC of 2.26 L and a **D** + **E** model-predicted FVC of 2.41 L. FEV_1_: a male patient underwent left upper lobectomy for squamous cell carcinoma, with a ground-truth FEV_1_ of 2.16 L and a **D** + **E** model-predicted FEV_1_ of 2.33 L. Grad-CAM heatmaps highlight the spatial regions that contributed most to the model’s predictions. The slice in each view corresponds to the location with the highest cumulative Grad-CAM activation along the respective anatomical axis. Representative axial (**a**, **c**) and coronal (**b**, **d**) views are shown. Panel i shows the original CT image of a representative patient; panel ii shows the corresponding Grad-CAM heatmap; panel iii shows the heatmap overlaid on the CT image; and panel iv shows the averaged attention map generated across all patients after resampling to a common input space. Warmer colors (red) indicate higher relevance, and cooler colors (blue) indicate lower relevance. FEV_1_, Forced expiratory volume in 1 s; FVC, Forced vital capacity

The best-performing model, **D** + **E**, is a hybrid deep- and machine-learning model. SHAP analysis was conducted to evaluate feature importance, and the top 20 features were visualized, as shown in Additional file 1, Supplementary Fig. S1. To further interpret these high-dimensional deep features, which lack the intrinsic interpretability of conventional radiomic features, Grad-CAM was used to visualize the regions of interest for representative patients (Fig. 5). The attention maps demonstrated that the primary focus in the postoperative FVC and FEV_1_ prediction models was located in the inferior lung regions adjacent to the diaphragm on axial views. In the FVC model, attention was concentrated on the central paravertebral soft tissues and adjacent lung fields. Coronal views also indicated a prominent focus in the right paravertebral region below the T12 vertebra, with another notable area of attention in the left upper lobe. In contrast, the FEV_1_ model showed that attention was distributed across the bilateral lung bases and surrounding diaphragmatic regions. Coronal views revealed an additional region of interest in the left lung, particularly in the lower lobe.

## Discussion

In this study, we developed a hybrid framework that coupled deep learning–based feature extraction with machine-learning regression to predict postoperative pulmonary function from preoperative CT. Compared with the clinical baseline model, conventional radiomics pipelines, and end-to-end deep learning, the I3D-based transfer-learning feature extractor combined with elastic net regression achieved superior performance. This indicated that hybrid modeling can better capture imaging patterns that are relevant to pulmonary functional outcomes. These findings support the use of transfer-learned 3D imaging representations as compact, task-oriented inputs for small-sample medical imaging problems.

Traditional approaches for estimating postoperative function rely on segment counting or resection-volume surrogates [16–24]. However, the segment-counting method is generally limited to lobectomy or segmentectomy and is less suitable for smaller resections such as wedge resection. CT-based volumetric approaches require both pre- and postoperative scans, limiting their use for true preoperative prediction. These methods also often rely on preoperative pulmonary function as a baseline, which can overestimate functional loss after anatomical resection. Moreover, postoperative recovery is not purely proportional to the anatomical extent of resection. Jeon et al [39] showed that functional recovery is lobe-dependent and often exceeds simple predictions, thus underscoring the role of lobe-specific physiology and compensatory mechanisms. Building on this rationale, we supplemented the imaging features with two categorical clinical variables, resection location and type, in addition to sex, age, height, and weight. Unlike counting or volumetric rules, these categorical factors provide a simple and flexible way to encode the surgical context that may not be fully captured by preoperative CT, while avoiding strong assumptions about proportional functional loss.

Studies have shown that a staged modeling strategy combining “deep features  + classical regression” can improve performance on small datasets [40–42]. For example, Zhang et al [42] transferred a neural network trained on 888 non-small cell lung cancer patients to a pancreatic ductal adenocarcinoma cohort (68 training, 30 test), using the network as a feature extractor and feeding the features into a random forest for prognostic prediction. This approach closely aligns with the staged modeling strategy used herein. A comparison of the five models revealed that the staged **D** + **E** model significantly outperformed the other approaches (external test set: *R*^2^ = 0.499 and CCC = 0.707 for FVC; *R*^2^ = 0.418 and CCC = 0.729 for FEV_1_). Several observations were made. Compared to **C** + **E**, clinical features alone showed a good correlation but limited agreement, likely due to the absence of baseline pulmonary function data, which can be supplemented by transfer-learned deep imaging features from a pre-trained pulmonary function prediction model. Compared with **R** + **E**, transfer-learned deep features appear to encode richer and more task-relevant 3D structures than handcrafted radiomics. In addition, the generalizability of the **R** + **E** model was limited in predicting postoperative FEV_1_. This might have been partly attributable to differences in baseline pulmonary function distributions between the internal and external cohorts. Specifically, the external cohort contained a higher proportion of patients with FEV_1_%pred < 80% and lower FEV_1_/FVC ratios despite comparable absolute FEV_1_ values. Compared with **D**, using a pre-trained and fine-tuned backbone purely as a feature extractor yields more stable representations when the data are limited, thereby mitigating the overfitting risks inherent in end-to-end optimization. Compared to **Res**, I3D initialization conferred a clear advantage over training from scratch, improving data efficiency and downstream accuracy, while avoiding substantial computational costs. We also compared the performance of the hybrid model with that of the conventional segment-counting method in a subset of the external test cohort. The predictive performance of the **D** + **E** model was significantly better than that of the segment-counting method, as indicated by permutation and Wilcoxon signed-rank tests. However, this analysis was exploratory and should be interpreted cautiously given the small sample size.

In addition to continuous regression metrics, we performed an exploratory threshold-based analysis using a clinically relevant cutoff of 80% predicted. The model had moderate discriminatory ability, but the limited number of patients below the clinical thresholds in the external test set might have affected the robustness and generalizability of these estimates. Larger studies are warranted to further evaluate model performance in threshold-based clinical decision-making scenarios.

Model interpretability analysis provides insights into image regions and feature representations associated with model predictions. The SHAP results revealed that nine high-dimensional features, which lacked direct biological meaning, were shared among the top 20 most important features of the FVC and FEV_1_ models, suggesting that the network captured a small set of common high-impact representations. Grad-CAM revealed that the FVC model primarily focused on the lower thoracic region, particularly the paravertebral soft tissues around the T12 level, as well as the adjacent lung parenchyma and the left upper lobe. This distribution suggested that, in addition to pulmonary information, the model may incorporate information from the chest wall structures involved in respiratory mechanics. Associations between chest wall soft tissues and pulmonary function have been identified [43–48], in which the cross-sectional area of the T12-level musculature was a predictor of both chronic obstructive pulmonary disease and post-lung transplantation outcomes [47, 48]. Our preliminary methodological investigation (Additional file 1, Method S1) found the potential relevance of extra-pulmonary information. The performance of models using only lung parenchyma worsened and was comparable to full-chest inputs when chest wall structures were added. This finding suggested that thoracic soft tissues could provide complementary information for postoperative FVC prediction and might be related to factors such as respiratory muscle strength and chest wall compliance [49, 50]. In contrast, the FEV_1_ model showed attention in the bilateral lung bases and diaphragmatic regions, with additional involvement of the left lower lobe. In contrast, the FEV_1_ model focuses on the bilateral lung bases and diaphragmatic regions, with additional involvement of the left lower lobe. Previous studies have reported associations between diaphragmatic thickness or motion and pulmonary function, particularly FEV_1_, providing a possible physiological context for the observed attention in the diaphragmatic region [51, 52]. However, Grad-CAM identifies regions that contribute to model predictions rather than the underlying physiological mechanisms; therefore, these findings should be interpreted with caution. Collectively, SHAP and Grad-CAM enhanced transparency in the model decision-making process and highlighted candidate regions for deeper imaging phenotyping or targeted clinical inquiries.

This study has several strengths. First, the pipeline requires minimal input (routine preoperative CT and a small set of basic clinical variables) and does not require preoperative spirometry or detailed surgical planning, thereby favoring broad applicability. Second, by leveraging transfer learning, the approach achieves strong performance with modest computational demands, facilitating practical deployment. Third, the combined use of global feature attribution (SHAP) and spatial attention (Grad-CAM) improves interpretability, potentially promoting clinician confidence and adoption.

This study has several limitations. First, although this was a multicenter study and an independent external test set was included, the overall sample size remained relatively small. This is primarily because postoperative PFTs are not part of routine follow-up and were only implemented when clinically indicated, leading to substantial and non-random attrition of eligible patients. This might have limited the robustness and generalizability of our findings. Future studies with larger prospective multicenter cohorts and standardized follow-up assessments are required to further validate the model and enhance its clinical applicability. In addition, the clinical features included in this study were relatively limited, which may have constrained the model's performance. Future studies could further optimize the model by incorporating additional relevant features.

In conclusion, the proposed hybrid model accurately predicted postoperative pulmonary function without requiring preoperative spirometry or detailed surgical planning, highlighting its potential for clinical application. The fine-tuned deep learning feature extractor effectively captured valuable features from the preoperative CT images, which were then used in downstream machine learning regression models. Interpretability analyses suggested that postoperative FVC predictions emphasized the inferior and paravertebral lung regions, whereas FEV_1_ predictions engaged the bilateral lung bases and diaphragmatic areas. These results highlight the need for prospective multicenter studies to determine the clinical value of this model for surgical decision-making and patient counseling.

## Supplementary information

**Additional file 1: Supplementary Table S1.** Radiomics features selected to establish the R+E model for predicting FVC (n=10) and FEV_1_ (n=9). **Supplementary Table S2.** Threshold-based classification performance of the D+E model in the external test set. **Supplementary Figure S1.** Model interpretation using SHAP visualizations for (a) FVC and (b) FEV_1_. The SHAP summary plots show the top 20 most important features, ranked by their impact on the model output. Each dot represents a single sample, with the color indicating the feature value (red: high; blue: low). The position along the x-axis reflects the magnitude and direction of the feature contribution.

## Data Availability

The datasets used and/or analyzed during the current study are available from the corresponding author on reasonable request.

## Abbreviations

CCC: Concordance correlation coefficient
CT: Computed tomography
FEV_1_: Forced expiratory volume in 1 s
FVC: Forced vital capacity
I3D: Inflated 3D ConvNet
MAE: Mean absolute error
MSE: Mean squared error
Pearson * r*: Pearson correlation coefficient
PFT: Pulmonary function test
*R*^2^: *R*-squared
RMSE: Root mean squared error
SHAP: SHapley Additive exPlanations
