# Supplementary material for: A transfer learning-based hybrid deep- and machine-learning regression approach for predicting the postoperative pulmonary function
Source: Eur Radiol Exp. 2026 Sep 30;10:146. doi: 10.1186/s41747-026-00821-z (PMC13627543; doi:10.1186/s41747-026-00821-z)
Supplement: Supplementary file 1 [file 41747_2026_821_MOESM1_ESM.pdf]

A Transfer Learning-Based Hybrid Deep- and Machine-Learning Regression Approach for Predicting the Postoperative Pulmonary Function

ELECTRONIC SUPPLEMENTARY MATERIAL

Method S1. Pretraining Dataset and Model Development

The pretraining dataset was obtained from a separate retrospective cohort at Huadong Hospital, Fudan University, between January 2018 and October 2023. This cohort was independent of the datasets used for model development and evaluation in the present study, with no patient-level overlap. The Ethics Committee of Huadong Hospital, Fudan University approved this study (Approval no: 20220026), and waived the requirement for written informed consent.

Patients were included if paired pulmonary function tests (PFT) and chest CT data were available, defined as a time interval of less than two weeks between CT acquisition and PFT examination. Additional inclusion criteria were no prior pulmonary surgery and high-quality images suitable for automated analyses. A total of 1,251 patients were ultimately included for model pre-training.

All CT images were retrieved from the institutional Picture Archiving and Communication System (PACS) in Digital Imaging and Communications in Medicine (DICOM) format and converted into a neuroimaging-compatible format (.nii.gz) using Python (version 3.10) and SimpleITK (version 2.3.1). Lung boundaries were manually reviewed to identify superior and inferior slice indices, and image volumes were cropped using Nibabel (version 5.1.0) to ensure that all slices contained lung tissue (Image_1).

TotalSegmentator (version 2.4.0) was used to generate voxel-wise labels

with 117 predefined anatomical structures for anatomical segmentation. Three input representations were constructed based on these segmentations:

Image_1: Full chest CT including all anatomical structures.

Image_2: Exclusion of heart, major vessels, skeletal structures, and abdominal organs, retaining lung and thoracic soft tissue.

Image_3: Lung-only representation including pulmonary lobes and trachea.

These input strategies were explored during model development as a preliminary methodological investigation and were not used for the primary model selection. The final pretraining model was trained using Image_1 (full chest CT input) as the default configuration.

The deep learning architecture was based on an improved I3D backbone. The network consisted of a global pooling layer (1,024 units), a fully connected layer (500 units), and a final linear output layer (1 unit). The model was optimized using mean squared error loss to minimize the discrepancy between the predicted and ground-truth pulmonary function values (FVC and FEV_1). The model implementation was performed using Keras and TensorFlow (version 2.8.0) on an NVIDIA GeForce RTX 3060 (12GB GPU).

Method S2. Radiomics + Elastic Net (R+E)

All images were downloaded from the PACS of Huadong Hospital and Heilongjiang Provincial Hospital in DICOM format. The images were imported into a 3D Slicer (version 5.4.0; loaded with the LungCTSegmenter extension), and the left lung, right lung, and large airway regions were semi-automatically labeled (left lung = 1, right lung = 2, and large airway = 3) and exported as NRRD format files. For the current study, only whole-lung tissue masks were required; therefore, the label values were adjusted by changing labels 2 to 1 and 3 to 0. This resulted in a single mask file representing the entire lung tissue (1 for lung tissue and 0 for nonlung tissue).

The processed masks were then used in Python (version 3.9.7) using the PyRadiomics package (version 3.1.0) to extract 1,218 radiomics features, including first-order statistics, texture, and shape features. To reduce multicollinearity, features with pairwise Pearson correlation coefficient > 0.8 (1,153 features) and variance inflation factor > 5 (34 features) were excluded. The remaining 31 features were further refined using stepwise regression to identify features significantly associated with FVC (10 features) or FEV_1 (nine features), as shown in **Supplementary Table S1**. These selected features were then used to build elastic net regression models, following the same approach as the D+E model described in the main text.

Method S3. 3D ResNet18 (Res)

The image preprocessing for Method 2 was identical to that used for the D and D+E models, including 12-bit normalization, lung slice selection, and resizing to 140 × 178 × 180 pixels. The model was based on a 3D ResNet18 architecture, with the final layers configured similarly to D and D+E. The outputs of the original penultimate layer (dense, 256 units) were concatenated with six standardized clinical features, followed by a dense layer (64 units) and a linear regression layer (1 unit). The model was implemented in PyTorch (version 2.7.1+cu128) using Python (version 3.10), and trained on an NVIDIA GeForce RTX 5070 Ti Laptop (12 GB) GPU for 50 epochs.

Supplementary Table S1. Radiomics features selected to establish the R+E model for predicting FVC (n=10) and FEV_1 (n=9).

Features NO	FVC	Features NO	FEV_1
1	original_firstorder_Energy	1	original_shape_Flatness
2	log-sigma-3-0-mm-3D_glrlm_LongRunLowGrayLevelEmphasis	2	original_firstorder_Energy
3	log-sigma-4-0-mm-3D_glcm_Imc2	3	log-sigma-3-0-mm-3D_glrlm_LongRunLowGrayLevelEmphasis
4	log-sigma-4-0-mm-3D_glcm_Idmn	4	log-sigma-4-0-mm-3D_glcm_Idmn
5	log-sigma-5-0-mm-3D_firstorder_Maximum	5	log-sigma-4-0-mm-3D_glszm_SizeZoneNonUniformityNormalized
6	wavelet-LLH_firstorder_Skewness	6	log-sigma-5-0-mm-3D_firstorder_Maximum
7	wavelet-LHH_firstorder_Mean	7	wavelet-LLH_firstorder_Skewness
8	wavelet-HLL_firstorder_Skewness	8	wavelet-LHL_firstorder_Skewness
9	wavelet-HLH_firstorder_Mean	9	wavelet-HHH_glszm_SizeZoneNonUniformityNormalized
10	wavelet-HHH_glcm_Idmn		

Supplementary Table S2. Threshold-based classification performance of the D+E model in the external test set.

Outcome	Cutoff	AUC	Accuracy	Sensitivity	Specficity	PPV	NPV
FVC%pred	<80%	0.745	0.645	0.909	0.500	0.500	0.909
FEV_1%pred	<80%	0.758	0.742	0.600	0.875	0.818	0.700

AUC=the area under the receiver operating characteristic curve; FEV_1=forced expiratory volume in 1 second; FVC=forced vital capacity; NPV=negative predictive value; PPV=positive predictive value.

Supplementary Figure S1. Model interpretation using SHAP visualizations for (a) FVC and (b) FEV_1.

a

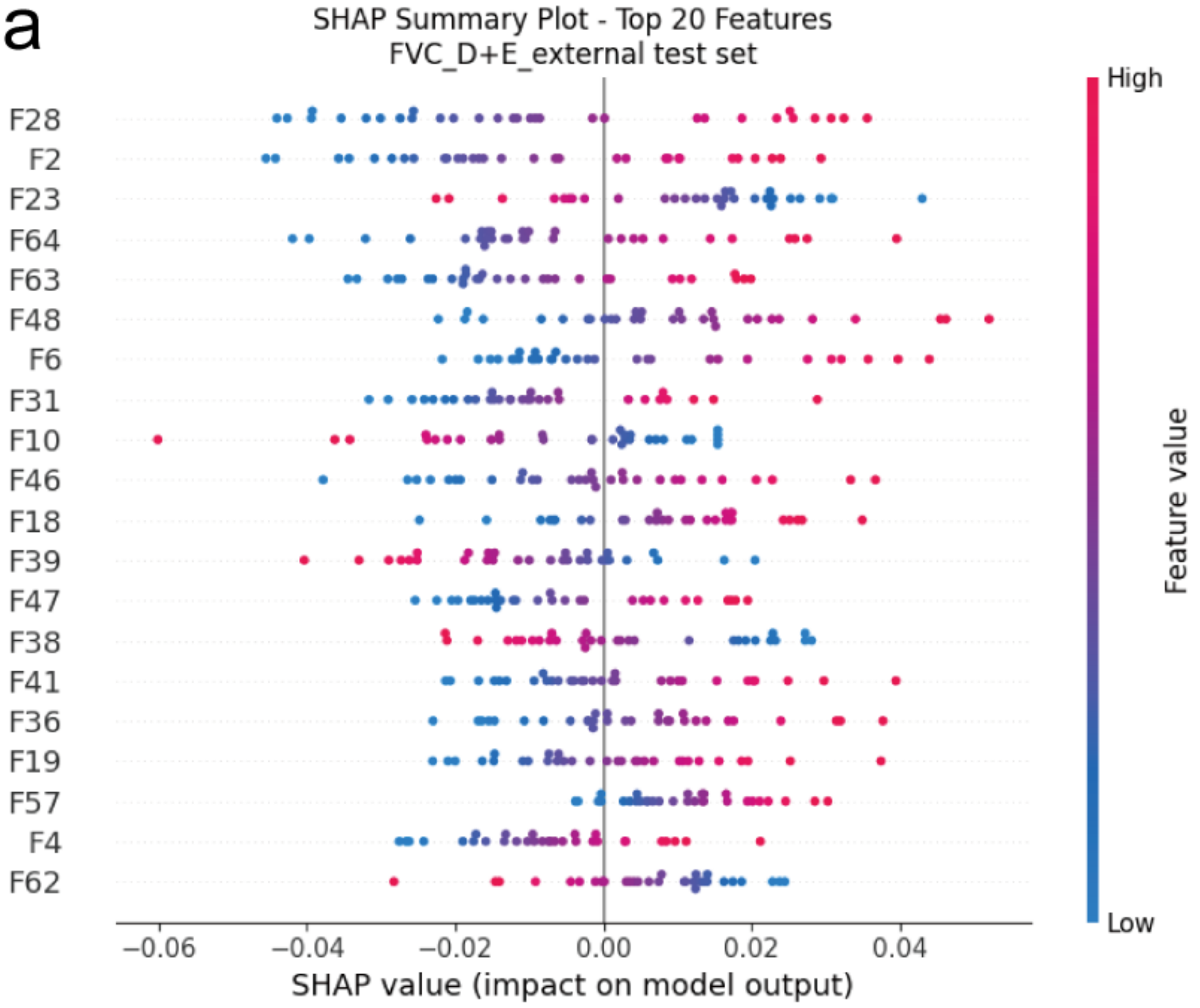

b

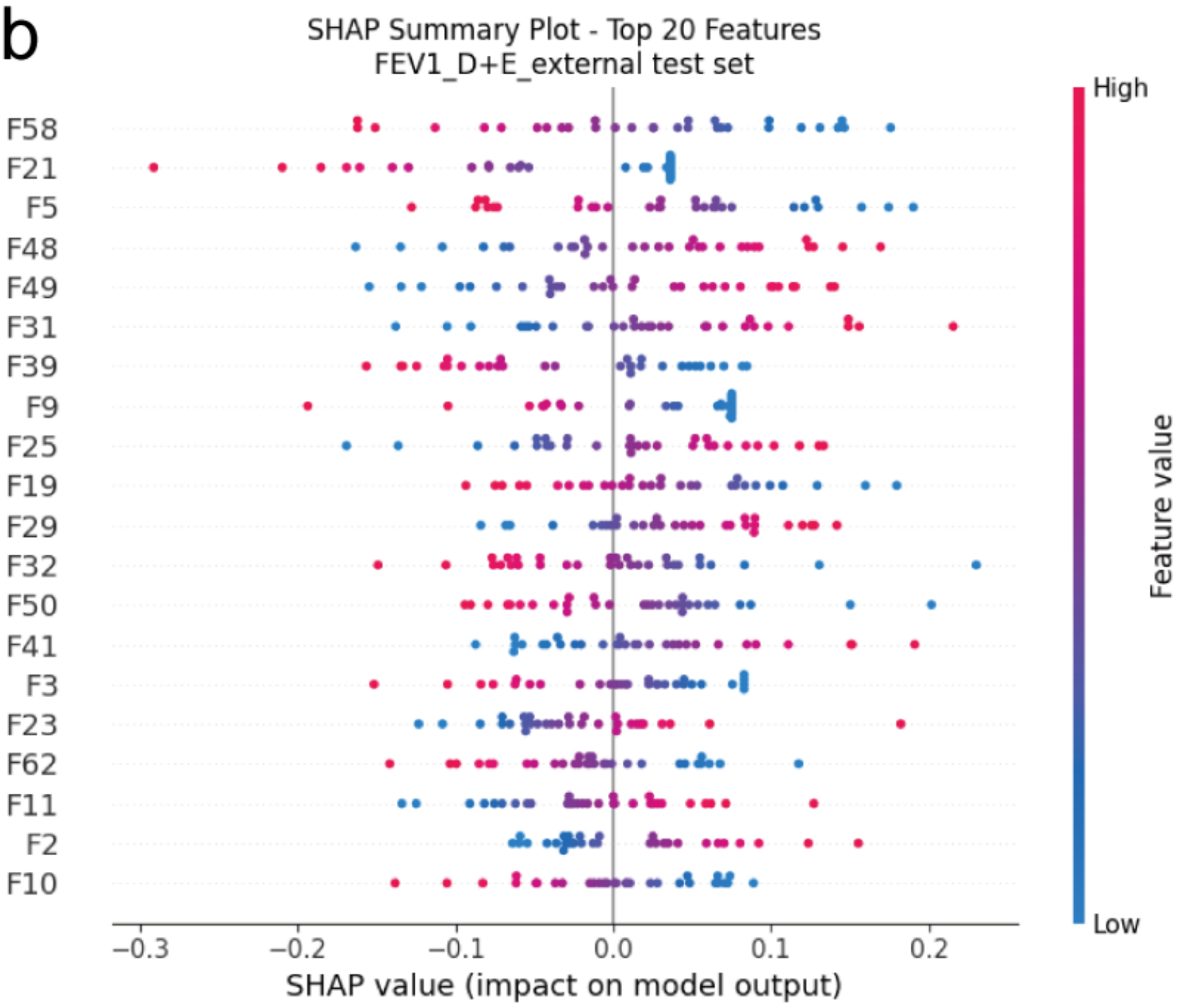

The SHAP summary plots show the top 20 most important features, ranked by their impact on the model output. Each dot represents a single sample, with the color indicating the feature value (red: high; blue: low). The position along the x-axis reflects the magnitude and direction of the feature contribution. FEV_1=forced expiratory volume in 1 second; FVC=forced vital capacity; SHAP=SHapley Additive exPlanations.
